# Effect of Preoperative Oral Antibiotics and Mechanical Bowel Preparations on the Intestinal Flora of Patients Undergoing Laparoscopic Colorectal Cancer Surgery: A Single-Center Prospective Pilot Study

**DOI:** 10.7759/cureus.52959

**Published:** 2024-01-25

**Authors:** Sho Fujiwara, Kenji Kaino, Kazuki Iseya, Nozomi Koyamada, Tatsuya Nakano

**Affiliations:** 1 Department of Surgery, Iwate Prefectural Chubu Hospital, Kitakami, JPN; 2 Department of Surgery, Columbia University Irving Medical Center, New York, USA; 3 Department of Surgery, Mito Medical Center, Ibaraki, JPN; 4 Department of Surgery, Iwate Prefectural Ofunato Hospital, Ofunato, JPN

**Keywords:** surgical site infection, laparoscopic surgery, intracorporeal anastomosis, colorectal cancer, bowel preparation

## Abstract

Introduction: In the last few decades, considerable progress has been made in controlling surgical site infections (SSIs) using a combination of mechanical and oral antibiotic bowel preparation. However, the number of bacteria present after bowel preparation has not been clarified. In this study, we investigated the bacterial cultures of intestinal fluid samples from patients undergoing laparoscopic surgery for colorectal cancer after preoperative bowel preparation.

Methods: This prospective observational study was designed as a pilot study at a single center. We enrolled 25 consecutive patients who underwent laparoscopic surgery for colorectal cancer between March 2021 and February 2022 at our institution.

Results: The rate of bacterial culture positivity was 56.0%. The most abundant bacterium was *Escherichia coli *(44.0%). The positivity rates for *E. coli* on the right and left sides were 54.5% and 35.7%, respectively (*P *= 0.60). Moreover, there was a significant relationship between a low American Society of Anesthesiologists Physical Status score and *E. coli* positivity on the right side (*P *= 0.031). In the left-sided group, female sex and large tumor size were significantly associated with *E. coli* positivity (*P *= 0.036 and 0.049, respectively). Superficial SSI occurred in the patient in the left-sided group, but *E. coli* was negative.

Conclusion: This study emphasizes the importance of understanding intestinal fluid contamination and its relationship to infection risk. Future prospective multicenter studies should be conducted to determine the association between intestinal bacteria and different types of preoperative preparation.

## Introduction

Recently, the rapid development of laparoscopic colorectal cancer surgery has enabled surgeons to perform total intracorporeal anastomosis (IA) [1,2]. Colorectal cancer surgery is associated with a higher risk of surgical site infections (SSIs) than other gastrointestinal surgeries [3]. More than 100 trillion bacterial cells are present in the human gastrointestinal tract [4,5]. There has been an increasing research interest in SSIs, especially among surgeons performing IA [6]. Moreover, considerable attention has been paid to preoperative bowel preparations, such as mechanical bowel preparation (MBP) with laxatives and oral antibiotic bowel preparation (OABP) [7-9].

Previous studies have demonstrated the advantages of IA [2,6,10-12], but IA can cause unintended contamination of intestinal fluid during anastomosis, and these studies have not addressed the following points. First, although previous findings support the importance of IA over extracortical anastomosis (EA) in SSI control, the mechanism by which intestinal fluid is contaminated remains unclear. Second, even if surgeons carefully avoid contamination by intestinal fluid, the risk of contamination is not eliminated, and the characteristics of patients with bacterial contamination remain unclear.

In the last few decades, progress has been made in controlling SSIs by utilizing OABP and MBP [7,8,13]. Several recent studies have shown that SSIs, including intra-abdominal abscesses, are less common with IA than with EA [6,12]. Most SSIs after colorectal surgery are caused by Escherichia coli, Enterococcus faecalis, Bacteroides fragilis, Enterobacter cloacae, or Pseudomonas aeruginosa [14]. Thus, preoperative bowel preparation is performed before colorectal surgery [8,13]. The latest guidelines published by the World Health Organization [15], Enhanced Recovery After Surgery Society [16], Society of American Gastrointestinal and Endoscopic Surgeons [17], American Society of Colon and Rectal Surgeons [18], and American College of Surgeons [19] recommend a combination of MBP and OABP to reduce SSI risk.

Thus, we hypothesized that a combination of MBP and OABP eliminates bacteria from the bowel lumen, but the effectiveness differs between right-sidedness and left-sidedness of the tumor. This would contribute to a decrease in the incidence of SSI due to unintended abdominal contamination. However, the number of bacteria remaining after the combination of bowel preparation has not been clarified in recent laparoscopic colorectal surgeries. In the present study, we investigated bacterial cultures of intestinal fluid samples from patients undergoing laparoscopic surgery for colorectal cancer after preoperative combinational bowel preparation with IA and EA. Furthermore, we analyzed the association between patient characteristics and bacterial cultures based on the sidedness of tumor location.

## Materials and methods

Patient selection and study design

**Figure 1:**
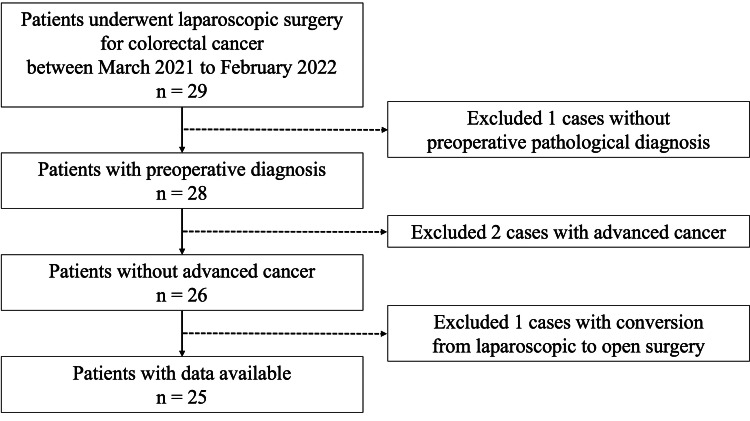
Flow diagram depicting the selection of the study participants.

This prospective, observational study was designed as a pilot study at a single center. We enrolled consecutive patients who underwent laparoscopic surgery for colorectal cancer between March 1, 2021, and February 28, 2022, at Iwate Prefectural Chubu Hospital, Japan. The patient selection process is illustrated in Figure 1. The inclusion criteria were as follows: colon or rectal cancer, operation completed using laparoscopic surgery, age > 20 years, no distant metastasis, no invasion into surrounding organs, pathological diagnosis based on preoperative biopsy examination, and no use of antibiotics within one month. The exclusion criteria were as follows: no definite diagnosis of colon or rectal cancer, bowel obstruction with cancer, unresectable factors, preoperative chemotherapy administration, stent placement, and history of allergy or other adverse reactions to the antimicrobial agents used in this study. The study was conducted in accordance with the ethical standards proposed in the Declaration of Helsinki, 1964, and approved by the institutional ethical board of the Ethics Committee of Iwate Prefectural Chubu Hospital (approval 11000793). Written informed consent was obtained from the study participants.

Preoperative bowel preparations

All patients underwent both MBP and OABP since 2020 at our institution as a standard practice in our hospital. OABP consisted of 500 mg metronidazole and 500 mg kanamycin after lunch and bedtime on the day before surgery. Intravenous cefmetazole (1 g) was administered every three hours during surgery. We used 24 mg of sennoside for right-sided colon cancer and 2 L of a polyethylene glycol solution for left-sided colorectal cancer.

Sample collection for intestinal fluid culture

Intestinal fluid samples were collected by swabbing the mucosal surface of the resected specimen around the tumor from the proximal to distal margin in the operating room after resection. Laboratory technicians who performed the culture tests were completely blinded to perioperative information, except for the location of the tumor. The technicians used both aerobic and anaerobic culture conditions. They also aimed to identify bacteria from the blinded samples.

Evaluation and quantification of cultural results

We classified the growth status of the identified organisms in the culture into five categories: 0 for no growth or minimal colony formation of only a few colonies in less than one-third of the plate, 1+ for less than one-third of the plate, 2+ for one-third to two-thirds of the plate, 3+ for more than two-thirds of the plate, and 4+ for the entire plate. The culture was defined as negative when the medium growth status was 0.

Clinical parameters

We evaluated the following clinical parameters to identify the risk factors for bacterial positivity after MBP and OABP: sex, age, tumor location, side of tumor location, body mass index, American Society of Anesthesiologists Physical Status (ASA-PS) classification, presence of a history of regular use of laxatives, current smoking, diabetes mellitus, anastomosis procedures, lymph node dissection, pathological differentiation, pathological Union for International Cancer Control (UICC) 8th edition TN factors and stages, tumor size, tumor markers (carcinoembryonic antigen (CEA) and carbohydrate antigen (CA)19-9), operative time, perioperative blood loss, hospitalization day, adverse effects of antibiotics, and postoperative complications (anastomotic leakage, intra-abdominal abscess, superficial SSI, and deep SSI).

Statistical analysis

We evaluated the association between the bacterial and clinical parameters. Categorical variables were analyzed using the chi-square and Fisher’s exact tests. Continuous variables were analyzed using a two-sided t-test and Mann-Whitney U test for parametric and nonparametric data, respectively. We additionally stratified the patients into two groups depending on tumor location, considering the difference in bacteria on the side of the colorectal tumor location. JMP Pro version 17 (SAS Institute Japan, Tokyo, Japan) was used for statistical analysis. Statistical significance was defined as P < 0.05.

## Results

Patient background

A total of 25 patients were enrolled in the study. The patient characteristics are summarized in Table 1. The median age was 67.0 years, 48.0% of the patients were female, and 56.0% of the tumors were located on the left side. The proportion of laxative users was 24.0%. Of the patients, 20.0% were anastomosed with IA, and 40.0% were anastomosed with a double-stapling system. The prevalence of superficial SSIs was 4.0%. No anastomotic leakage, deep SSI, or intra-abdominal abscess were observed.

**Table 1:** Characteristics of study participants IQR; Interquartile range, SD; Standard deviation, ASA-PS; American Society of Anesthesiologists Physical Status, UICC; Union for International Cancer Control, CEA; Carcinoembryonic antigen, CA19-9; Carbohydrate antigen, SSI; Surgical site infection

Characteristics			
Age years, median [IQR]	67.0 [57.4–79.5]	Pathological differentiation, No. (%)	
Sex (Female/Male), No. (%)	12/13 (48.0/52.0)	Poor	1 (4.0)
Tumor location, No. (%)		Moderate	6 (24.0)
Cecum	5(16.0)	High	18 (72.0)
Ascending colon	5 (20.0)	UICC T (8th), No. (%)	
Transverse colon	2 (8.0)	1	10 (40.0)
Descending colon	2 (8.0)	2	7 (28.0)
Sigmoid colon	5 (20.0)	3	8 (32.0)
Rectum	7 (28.0)	UICC N (8th), No. (%)	
Sidedness (right/left), No. (%)	11/14 (44.0/56.0)	0	20 (80.0)
Body mass (index kg/m^2^), mean±SD	23.1 ± 3.2	1	5 (20.0)
ASA-PS, No. (%)		Tumor size (mm), median [IQR]	25.0 (19.0–30.0)
1	7 (28.0)	CEA (ng/mL), median [IQR]	2.8 (1.9–4.7)
2	9 (36.0)	CA19-9 (U/mL), median [IQR]	7.3 (2.8–12.9)
3	6 (24.0)	Operative time (min)	
4	3 (12.0)	Intraoperative blooding (mL)	
Laxative, No. (%)	3 (12.0)	Postoperative hospitalized stay (days)	
Smoker, No. (%)	10 (40.0)	Adverse effect of oral antibiotics, No. (%)	0 (0.0)
Diabetes mellitus, No. (%)	2 (8.0)	Anastomotic leakage, No. (%)	0 (0.0)
Anastomosis, No. (%)		Superficial SSI, No. (%)	1 (4.0)
Extracortical	10 (40.0)	Deep SSI, No. (%)	0 (0.0)
Intracortical	5 (20.0)	Intraabdominal abscess, No. (%)	0 (0.0)
Double stapling system	10 (40.0)		
Lymph node dissection, No. (%)			
D2	13 (52.0)		
D3	12 (48.0)		

Results of microbial culture

Table 2 summarizes the culture results of the samples, and Table 3 summarizes the prevalence of the five major bacteria causing SSIs: Escherichia coli, Enterococcus faecalis, Bacteroides fragilis, Enterobacter cloacae, and Pseudomonas aeruginosa. The rate of bacterial culture positivity was 56.0%. There were no significant differences in sidedness. Further analysis revealed that the most abundant bacterium was E. coli (44.0%). The positivity rates of E. coli on the right and left sides were 54.5% and 35.7%, respectively. Enterococcus faecalis was detected in 21.4% and 9.1% of patients with left-sidedness and right-sidedness, respectively. However, this difference was not significant (P = 0.60). These findings indicated that E. coli was the predominant bacterial species on both sides.

**Table 2:** Identification of bacterial culture species and their colorectal locations

Location	Characteristic bacteria
Cecum	Bacillus, Bacteroides fragilis, Candida albicans, Escherichia coli, Klebsiella pneumonia, Serratia marcescens, Staphylococcus aureus, Streptococcus bovis
Ascending colon	Bacillus, Candida albicans, Enterobacter cloacae, Enterococcus faecalis, Enterococcus raffinosus, Escherichia coli, Klebsiella oxytoca, Klebsiella pneumonia, Pseudomonas aeruginosa
Transverse colon	Bacillus, Escherichia coli, Klebsiella oxytoca, Pseudomonas aeruginosa, Streptococcus bovis
Descending colon	Enterococcus avium, Enterococcus faecalis, Escherichia coli, Klebsiella oxytoca, Morganella morganii, Pseudomonas aeruginosa, Streptococcus bovis
Sigmoid colon	Enterococcus faecalis, Klebsiella oxytoca, Streptococcus bovis
Rectum	Bacillus, Edward, Escherichia coli, Group G, Klebsiella oxytoca, Klebsiella pneumonia

**Table 3:** Positive rate of microbial culture: major risk species for surgical site infection *P < 0.05 is statistically significant

Bacteria	Total	Right-sidedness	Left-sidedness	P-value
	No. (%)	No. (%)	No. (%)	
Any five bacteria	14/25 (56.0)	7/11 (63.6)	7/14 (50.0)	0.69
Escherichia coli	11/25 (44.0)	6/11 (54.5)	5/14 (35.7)	0.43
Enterococcus faecalis	4/25 (16.0)	1/11 (9.1)	3/14 (21.4)	0.60
Bacteroides fragilis	1/25 (4.0)	1/11 (9.1)	0/14 (0.0)	0.44
Enterobacter cloacae	1/25 (4.0)	1/11 (9.1)	0/14 (0.0)	0.44
Pseudomonas aeruginosa	2/25 (8.0)	1/11 (9.1)	1/14 (7.1)	1.00

Clinical factors for cultural positivity

To investigate the association between the clinical background of the patients and culture positivity for E. coli, we classified them into two groups: right and left sides (Table 4). There was a statistically significant relationship between low ASA-PS score and E. coli positivity on the right side (P = 0.031). However, no significant difference was observed between sexes and E. coli positivity (P = 0.57). In the left-sided group, female sex and large tumor size were significantly associated with E. coli positivity (P = 0.036 and 0.049, respectively). Superficial SSI occurred in the patient in the left-sided group, but E. coli was negative.

**Table 4:** Associations between Escherichia coli positivity and cohort characteristics *P < 0.05 is statistically significant IQR; Interquartile range, SD; Standard deviation, ASA-PS; American Society of Anesthesiologists Physical Status, UICC; Union for International Cancer Control, CEA; Carcinoembryonic antigen, CA19-9; Carbohydrate antigen, SSI; Surgical site infection

Characteristics	Right-sidedness			Left-sidedness		
	Positive	Negative	P-value	Positive	Negative	P-value
	(n = 6)	(n = 5)		(n = 5)	(n = 9)	
Age years, median [IQR]	73.5 [60.8–83.5]	82.0 [79.5–83.5]	0.15	54.0 [46.0–61.5]	60.0 [57.5–69.0]	0.066
Sex (Female), No. (%)	4 (66.7)	2 (40.0)	0.57	4 (80.0)	2 (22.2)	0.036*
Body mass index (kg/m2), mean±SD	22.0 ± 3.8	22.5±0.9	0.52	24.0 ± 2.4	23.7 ± 4.2	0.59
ASA-PS, No. (%)			0.031*			0.78
1	2 (33.3)	0 (0.0)		3 (60.0)	2 (22.2)	
2	3 (50.0)	1 (20.0)		1 (20.0)	4 (44.4)	
3	0 (0.0)	3 (60.0)		1 (20.0)	2 (22.2)	
4	1 (16.7)	1 (20.0)		0 (0.0)	1 (11.1)	
Laxative, No. (%)	0 (0.0)	2 (40.0)	0.12	1 (20.0)	1 (11.1)	1.00
Smoker, No. (%)	3 (50.0)	2 (40.0)	1.00	1 (20.0)	4 (44.4)	0.58
Diabetes mellitus, No. (%)	0 (0.0)	1 (20.0)	1.00	1 (20.0)	0 (0.0)	0.36
Lymph node dissection, No. (%)			1.00			1.00
D2	3 (50.0)	2 (40.0)		3 (60.0)	5 (55.6)	
D3	3 (50.0)	3 (60.0)		2 (40.0)	4 (44.4)	
Pathological differentiation, No. (%)			0.061			0.58
Poor	0 (0.0)	1 (20.0)		0 (0.0)	0 (0.0)	
Moderate	0 (0.0)	2 (40.0)		2 (40.0)	2 (22.2)	
High	6 (100)	2 (40.0)		3 (60.0)	7 (77.8)	
UICC (8th) Stage, No. (%)			0.89			0.49
1	3 (50.0)	3 (60.0)		4 (80.0)	5 (55.6)	
2	2 (33.3)	1 (20.0)		0 (0.0)	2 (22.2)	
3	1 (16.7)	1 (20.0)		1 (20.0)	2 (22.2)	
UICC T (8th), No. (%)			0.24			0.33
1	2 (33.3)	0 (0.0)		2 (40.0)	6 (66.7)	
2	2 (33.3)	4 (80.0)		1 (20.0)	0 (0.0)	
3	2 (33.3)	1 (20.0)		2 (40.0)	3 (33.3)	
UICC N (8th), No. (%)			0.89			0.92
0	5 (83.3)	4 (80.0)		4 (80.0)	7 (77.8)	
1	1 (16.7)	1 (20.0)		1 (20.0)	2 (22.2)	
Tumor size (mm), median [IQR]	21.0 [16.8–33.8]	20.0 [15.0–42.5]	0.93	30.0 [25.0–42.5]	20.0 [10.0–29.0]	0.049*
CEA ng/mL, median [IQR]	4.5 [1.4–11.8]	3.3 [1.6–9.5]	0.71	1.9 [1.1–4.7]	2.3 [2.0–2.8]	0.14
CA19-9 U/mL, median [IQR]	7.7 [4.5–37.1]	12.9 [4.1–23.1]	0.78	12.3 [2.7–14.0]	3.2 [2.6–7.1]	0.23
Operative time (min), median [IQR]	252.0 [207.3–304.3]	222.0 [155.0–271.0]	0.41	233.0 [195.0–273.5]	204.0 [185.0–291.0]	1.00
Blood loss (mL), mean±SD	13.2 ± 9.2	19.0 ± 16.4	0.59	38.0 ± 12.9	18.8 ± 9.5	0.79
Postoperative hospitalized stay, (day) median [IQR]	6.5 [5.0–10.0]	6.0 [4.5–9.0]	0.64	6.0 [0.0–7.0]	7.0 [6.5–9.5]	0.065
Superficial SSI, No. (%)	0 (0.0)	0 (0.0)	1.00	0 (0.0)	1 (11.1)	1.00

Although we investigated the association between the clinical background of patients and culture positivity for any bacteria, no statistically significant relationships were observed (Table 5).

**Table 5:** Associations between any bacteria positivity and cohort characteristics *P<0.05 is statistically significant IQR; Interquartile range, SD; Standard deviation, ASA-PS; American Society of Anesthesiologists Physical Status, UICC; Union for International Cancer Control, CEA; Carcinoembryonic antigen, CA19-9; Carbohydrate antigen, SSI; Surgical site infection

Characteristics	Right-sidedness			Left-sidedness		
	Positive	Negative	P-value	Positive	Negative	P-value
	(n = 7)	(n = 4)		(n = 7)	(n = 7)	
Age years, median [IQR]	75.0 [67.0–83.0]	82.0 [78.8–83.8]	0.30	57.0 [47.0–64.0]	60.0 [58.0–70.0]	0.18
Sex (Female), No. (%)	4 (57.1)	2 (50.0)	0.82	4 (57.1)	2 (28.6)	0.59
Body mass (index kg/m^2^), mean±SD	22.3 ± 3.5	22.2 ± 0.3	0.92	22.8 ± 2.9	24.9 ± 4.0	0.27
ASA-PS, No. (%)			0.44			0.55
1	2 (28.6)	0 (0.0)		3 (42.9)	2 (28.6)	
2	3 (42.9)	1 (25.0)		2 (28.6)	3 (42.9)	
3	1 (14.3)	2 (50.0)		2 (28.6)	1 (14.3)	
4	1 (14.3)	1 (25.0)		0 (0.0)	1 (14.3)	
Laxative, No. (%)	1 (14.3)	1 (25.0)	1.00	1 (14.3)	1 (14.3)	1.00
Smoker, No. (%)	4 (57.1)	1 (25.0)	0.55	3 (42.9)	2 (28.6)	1.00
Diabetes mellitus, No. (%)	1 (14.3)	0 (0.0)	1.00	1 (14.3)	0 (0.0)	1.00
Lymph node dissection, No. (%)			0.30			1.00
D2	4 (57.1)	1 (25.0)		4 (57.1)	4 (57.1)	
D3	3 (42.9)	3 (75.0)		3 (42.9)	3 (42.9)	
Pathological differentiation, No. (%)			0.31			1.00
Poor	0 (0.0)	1 (25.0)		0 (0.0)	0 (0.0)	
Moderate	1 (14.3)	1 (25.0)		2 (28.6)	2 (28.6)	
High	6 (85.7)	2 (50.0)		5 (71.4)	5 (71.4)	
UICC (8th) Stage, No. (%)			0.44			0.80
1	3 (42.9)	3 (75.0)		5 (71.4)	4 (57.1)	
2	2 (28.6)	1 (25.0)		1 (14.3)	1 (14.3)	
3	2 (28.6)	0 (0.0)		1 (14.3)	2 (28.6)	
UICC T (8th), No. (%)			0.44			0.35
1	2 (28.6)	0 (0.0)		3 (42.9)	5 (71.4)	
2	3 (42.9)	3 (75.0)		1 (14.3)	0 (0.0)	
3	2 (28.6)	1 (25.0)		3 (42.9)	2 (28.6)	
UICC N (8th), No. (%)			0.49			1.00
0	5 (71.4)	4 (100.0)		6 (85.7)	5 (71.4)	
1	2 (28.6)	0 (0.0)		1 (14.3)	2 (28.6)	
Tumor size (mm), median [IQR]	20 [18–30]	27.5 [12.5–46.3]	0.70	35 [25–35]	20 [10–28]	0.11
CEA (ng/mL), median [IQR]	4.3 [1.5–11.2]	5.7 [1.7–10.1]	0.93	1.9 [1.6–33]	2.3 [2.0–2.8]	0.61
CA19-9 (U/mL), median [IQR]	7.3 [4.3–9.4]	15.3 [7.6–25.9]	0.19	8.5 [2.8–13]	3.1 [2.5–5.7]	0.14
Operative time (min), median [IQR]	249.0 [210.0–304.0]	199.5 [144.0–275.3]	0.30	233.0 [168.0–277.0]	204.0 [186.0–282.0]	1.00
Blood loss (mL), mean±SD	14.9 ± 9.5	17.5 ± 18.5	0.80	31.6 ± 39.8	19.7 ± 13.2	0.48
Post operative hospitalized stay (day), median [IQR]	7.0 [5.0–11.0]	5.5 [4.3–6.8]	0.21	6.0 [0.0–7.0]	7.0 [7.0–9.0]	0.10
Superficial SSI, No. (%)	0 (0.0)	0 (0.0)	1.00	0 (0.0)	1 (14.3)	1.00

## Discussion

We found that the culture positivity rate of the intestinal fluid samples was 56% after combination preparation, and most samples were positive for E. coli. Sex, tumor size, and ASA-PS scores were significantly associated with bacterial culture positivity. However, bacterial culture positivity was not associated with the occurrence of SSI. IA has been shown to reduce the risk of SSIs and intra-abdominal abscesses compared with EA [6,12]. While the combination of MBP and OABP for preoperative bowel preparation minimizes the risk of SSIs, little is known about the number of bacteria present in the intestinal fluid after preoperative bowel preparation [7,8]. The present study was designed to identify the bacterial species in the intestinal fluid after MBP and OABP. We collected intestinal fluid for cultures during laparoscopic colorectal surgery.

As suggested in previous studies, we found that the characteristics of cancer patients, including sex, are related to the bacterial flora of the intestinal mucosa [20-24]. Previous studies have reported that the abundance of Bacteroides is related to physical activity in athletes and gait speed in healthy elderly women [25,26]. Another study reported a negative correlation between Enterobacteriaceae and gait speed in Parkinson’s patients [27]. Furthermore, higher ASA-PS has been reported to be associated with resistance to certain antibiotics [14]. Hence, the ASA-PS score could be related to intestinal flora through many confounding factors. This may be because bacterial positivity did not affect the occurrence of SSIs.

We found that approximately 50% of intestinal fluid samples contained bacteria. To the best of our knowledge, no study has analyzed the culture of intestinal fluid after preoperative bowel preparation, as conducted in our study. In 1989, Sasaki et al. cultured 1 g of fecal samples after MBP and OABP [28]. They reported that MBP and OABP decreased the number of aerobic and anaerobic bacteria from 6.6±1.7 log10/g to 4.2±1.9 log10/g and 7.3±1.9 log10/g to 3.0±2.1 log10/g, respectively [28]. Approximately 24% of aerobic and 70% of anaerobic bacteria were below the detection limit [28]. Thus, considering these results, our results on the bacterial positivity rate after preparation are feasible. However, Sasaki et al. administered oral antibiotics at higher doses and for longer periods than those utilized by us herein. Therefore, our study is unique in that our preoperative preparations were consistent with the current real-world settings, suggesting that the current preparation is effective and safe.

Our study had several limitations. First, the sample size is small. Given that our findings were based on a limited number of patients, our results must be interpreted with caution. Although we could not make strong statements based on our research, we believe that patient characteristics can influence intestinal bacteria. Second, our study could not evaluate and compare the microbiomes without antibiotics. We need more data comparing the effects of antibiotics. Moreover, this study did not provide detailed data on bacterial identification. We investigated the bacterial culture positivity of intestinal fluid samples; however, recent studies on microbiomes have used 16S ribosomal RNA sequencing [29]. The obstruction and chemotherapy can influence these microbiomes. In this study, we excluded these patients to determine the patients' backgrounds. However, we need to investigate the microbiomes of these patients because recent advances in surgery enable us to perform minimally invasive surgery for these advanced patients. Further investigation that can reflect real-world clinical outcomes from a clinical perspective remains to be determined.

This study presents novel data on the bacterial characteristics found in the intestinal fluid of patients undergoing colorectal cancer surgery after MBP and OABP. Our findings can help advance the understanding of the influence of intestinal preparation and reduction of SSIs during IA. These can also give the fundamental data to optimize the appropriate MBP and OABP methods for colorectal cancer surgery. It may contribute to developing the strategy of preparation and reducing SSIs.

## Conclusions

Our study provides insight into the risk of SSIs and the safety of IA. Despite these limitations, our study serves as a starting point for discussions regarding intestinal fluid contamination and SSI control. Our results suggest that higher ASA-PS and larger tumor size may be risk factors for bacterial positivity. However, appropriate preparation may allow us to perform IA safely. Future prospective multicenter studies should investigate the association between intestinal bacteria and various types of preoperative preparation. Additionally, further studies are required to validate our findings.
